# Immunodominance hierarchy after seasonal influenza vaccination

**DOI:** 10.1080/22221751.2022.2135460

**Published:** 2022-11-04

**Authors:** Laura Sánchez-de Prada, Iván Sanz-Muñoz, Raúl Ortiz de Lejarazu, José María Eiros, Adolfo García-Sastre, Teresa Aydillo

**Affiliations:** a National Influenza Centre of Valladolid, Valladolid, Spain; b Department of Microbiology, Hospital Clínico Universitario de Valladolid, Valladolid, Spain; c Department of Microbiology, Icahn School of Medicine at Mount Sinai, New York, NY, USA; d Global Health and Emerging Pathogens Institute, Icahn School of Medicine at Mount Sinai, New York, NY, USA; e Department of Pathology, Molecular and Cell-Based Medicine, Icahn School of Medicine at Mount Sinai, New York, NY, USA; f Department of Medicine, Division of Infectious Diseases, Icahn School of Medicine at Mount Sinai, New York, NY, USA; g The Tisch Cancer Institute, Icahn School of Medicine at Mount Sinai, New York, NY, USA

**Keywords:** Immunodominance, hemagglutinin, influenza, vaccines, adjuvants

## Abstract

Current influenza vaccines elicit humoral immune responses against the haemagglutinin (HA) protein of influenza viruses. Different antigenic sites have been identified in the HA head as the main target of haemagglutination inhibition (HAI) antibodies (Sb, Sa, Cb, Ca1 and Ca2). To determine immunodominance (ID) of each site, we performed HAI assays against a panel of mutant viruses, each one lacking one of the classically defined antigenic sites and compared it to wild type (Wt). Agglutinating antibodies were measured before and after vaccination in two different regimens: Quadrivalent Influenza Vaccine (QIV) in young adults; or Adjuvanted Trivalent influenza Vaccine (ATIV) in elderly. Our results showed abs before vaccination were significantly reduced against all antigenic sites in the elderly and only against Sb and Ca2 in young adults compared to the Wt. Humoral response to vaccination was significantly reduced against all viruses compared to the Wt for the ATIV and only against Sb and Ca2 for the QIV. The strongest reduction was observed in all cases against Sb followed by Ca2. We concluded that ID profile was clearly dominated by Sb followed by Ca2. Additionally, the antibody response evolved with age, increasing the response towards less immunodominant epitopes of HA head. Adjuvants can positively influence ID hierarchy broadening responses towards multiple antigenic sites of HA head.

## Introduction

Seasonal influenza represents an important socioeconomic burden [1]. According to the World Health Organization (WHO), influenza epidemics affect 10–20% of the global population; and are responsible for approximately three to five million severe cases and 290,000–650,000 respiratory deaths each year [2,3]. Additionally, the economic impact has been estimated in a total annual burden of 6–14 billion euros in the European Union and 87.1 billion dollars in the United States alone [4,5]. The best strategy to address influenza epidemics is through annual vaccination campaigns. However, according to the Centers for Disease Control and Prevention, the effectiveness of the current vaccines is moderate ranging from 20 to 70% depending on the season [6]. On the other hand, influenza virus infection provides short-lasting strain-specific protection through neutralizing antibodies that impair viral attachment and membrane fusion [7]. The major target of the antibodies induced by vaccination and infection is the head of the haemagglutinin (HA), the major glycoprotein of the surface. One of the reasons for the poor effectiveness of vaccines, is that the error-prone polymerase of influenza A viruses causes amino acid substitutions that rapidly accumulate in the HA head, enabling immune evasion [8–10]. That phenomenon, known as antigenic drift, makes necessary to reformulate and re-administer vaccines yearly [11].

The HA protein contains two major extracellular domains, a plastic globular head, and a highly conserved stem. Antibodies against the stem are more cross-reactive and can bind different strains of the same phylogenetic group providing broad protection against severe outcomes [12]. However, the immune response to vaccination or infection generally promotes the generation of antibodies that bind to a limited number of immunodominant antigenic sites [13]. Those sites, located in the head of the HA, lead to strain-specific protection after exposure [14]. Classically, five antigenic sites in the head of the HA have been identified, characterized and defined as Sb, Sa, Cb, Ca1 and Ca2 [15,16]. The first two, are placed on the distal tip of each monomer; while Cb, Ca1 and Ca2 are placed proximally, near the stalk domain. The receptor binding site (RBS), where the attachment to sialic acids occurs, is located between Sb, Ca2 and Sa [8,16,17]. Several efforts have been made to characterize the ID hierarchy for influenza viruses. This is critical to fully understand antibody immunity to influenza viruses and guide the design of future improved influenza vaccines [18,19].

It has been previously shown that the antigenic sites in influenza H1 haemagglutinin display species-specific immunodominance (ID). Additionally, the antibody-mediated immune responses against the head domain of an H1 haemagglutinin differ in animals and humans after vaccination [18]. To quantify ID after influenza vaccination in humans, a panel of mutant viruses each lacking a classically defined antigenic site in the head of the HA protein of the pandemic-like H1 strain A/Michigan/45/2015 was used. We determined the ID of the mentioned antigenic sites in young adults and elderly after vaccination with a quadrivalent influenza vaccine (QIV) or adjuvanted trivalent influenza vaccine (ATIV), respectively. Our results suggest the existence of an age-related evolution of ID hierarchy with broader response towards all sites in the elderly. Additionally, adjuvants could play a role in broadening the response towards subdominant antigenic sites.

## Materials and methods

### Patient recruitment

A total of 162 individuals were recruited from vaccination programmes during the Influenza Vaccine Campaign (IVC) 2018 conducted by the Influenza Sentinel Surveillance Network of Castile and Leon (ISSNCyL) and the National Influenza Centre of Valladolid (Spain) to assess vaccine immunogenicity of the population. Those samples were used to perform ID studies at the Mount Sinai Hospital in New York (U.S.A). Serum was obtained by clinicians before influenza vaccination and 28 days after. Two seasonal influenza vaccines were used following the WHO recommendations for the Northern hemisphere; and contained: A/Michigan/45/2015 (H1N1)pdm09-like virus, A/Singapore/INFIMH-16-0019/2016 (H3N2)-like virus and B/Colorado/06/2017-like virus (B/Victoria/2/87 lineage) for ATIV and B/Phuket/3073/2013-like virus (B/Yamagata/16/88 lineage) for QIV. Following vaccine recommendations in Spain, subjects ≥ 65 years old received an ATIV (Chiromas, Seqirus) and subjects <65 years old received an QIV (Vaxigrip Tetra, Sanofi Pasteur).Written informed consent was obtained from participants. This research was performed according to the Declaration of Helsinki and approved by the Ethics Committee of East-Valladolid health area under the code PI 21-2314.

### Panel of virus

A panel of five recombinant viruses was generated: H1-ΔSa, H1-ΔSb, H1-ΔCa1, H1-ΔCa2, H1-ΔCb, were the classically defined H1 antigenic sites (Sb, Sa, Cb, Ca1 and Ca2) had been partially substituted with heterologous antigenic sites from either H5 or H13 HAs. Specifically, each mutant virus contained five or more amino acid substitutions within one antigenic site while the other four sites remained intact. The methods and description of the generation of these viruses have been previously published [18]. Viruses were kindly donated by Peter Palese. Viruses were cultured in allantoid fluid in 10-days-old embryonated chicken eggs and then haemagglutination assay was performed to confirm the growth of each virus before freezing at −80°C. To ensure the viruses had not suffered egg-adaptative mutations, they were sequenced before their use.

### Haemagglutination inhibition assay (HAI)

The presence of antibodies in serum samples was analysed following the WHO and the Influenza Surveillance Network for the surveillance of influenza viruses and vaccine efficacy protocol [20]. Beforehand to the HAI, 100 µl of each sample was treated with 300 µl of RDE (Receptor Destroying Enzyme; Denka Seiken, Tokyo, Japan) to eliminate non-specific inhibitors. This solution was incubated overnight (12–18 h) at 37°C in a water bath and then deactivated with 300 µl of 2.5% sodium citrate solution and diluted in 300 µl PBS to a work concentration of 1/10. Before performing HAI, each virus was titred and standardized to 8 haemagglutination units (8HU)/50 µl after thawing. Two-fold dilutions of 25 µl of each serum in 25 µl of HA buffer were conducted in 96-V-microwell plates, and then 25 µl of the virus was incorporated and incubated for 30 min at room temperature. Finally, 50 µl of turkey erythrocytes at 0.5% were added and incubated at 4°C for another 45 min. The HAI titre was defined as the highest dilution at which haemagglutination inhibition occurred. Assays were performed in triplicates and to ensure non-specific agglutinants were present a negative and a positive control were added to each sample.

### Statistical analysis

Population characteristics were compared using the chi-square test for categorical variables Mann–Whitney *U*-test for continuous variables. For HAI analysis, different parameters were calculated. Negative results in HAI were assumed as half of the detection threshold (1/5). The Geometric Mean Titre (GMT) and Confidence interval 95% (CI95%) were computed by taking the exponent (log10) of the mean and of the lower and upper limits of the CI95% of the log10-transformed values. Fold-induction (FI) was calculated dividing the post-/pre-vaccine GMT. Seroprotection rate (SPR) was calculated as percentage of individuals with antibody titres ≥ 1/40 and, seroconversion rate (SCR), as percentage of individuals showing at least a four-FI of pre-vaccination titres. To represent ID, we calculated two parameters: Fold induction rate (FIR) as “(FI mutant virus/FI Wt H1) × 100”, and HAI dominance index (DI) as the reduction of HAI titres before and after vaccination of mutant viruses compared its respective Wt H1(GMT Wt H1/GMT mutant virus) [18]. For quantitative variables, comparison of each virus against its Wt H1 was computed by repeated measures one-way Bonferroni’s ANOVA for multiple comparisons test with the Geisser–Greenhouse correction and, Student *T*-test was used for comparison between vaccines, and Friedman’s test with Dunńs correction for comparisons before and after vaccination. For categorical variables, comparison between each mutant virus and its cognate Wt H1 was computed with McNemar’s test with the continuity correction and, with chi-square when comparing vaccines. All reported *P*-values are based on two-tailed tests computed by SPSS vs27 (IBM, Armonk, NY, USA) and GraphPad Prism vs9 (GraphPad, San Diego, CA, USA), and taking statistical significance at the *P* < .05 value.

## Results

### Human cohorts

One hundred sixty-two individuals were recruited. Two different influenza-inactivated vaccines were applied according to age: a QIV in subjects of 28–64 years (46, 28.4%); and a MF59-ATIV in subjects ≥65 years old (116, 71.6%). Blood samples were collected on day 0 (pre-) and on day 28 after vaccination (post-). Age and sex of both cohorts are described in Table 1.

**Table 1.** Age and sex of QIV and ATIV cohorts.

	QIV	ATIV	*P*-value
*N*	46	116	
Age (mean, CI95)	52.3 (49.0–55.6)	78.6 (76.9–80.3)	<.0001***
Men (n, %)	19 (41.3%)	62 (53.45%)	.135

Two-tailed *P*-value was calculated using Mann–Whitney *T*-test for age and chi-square for sex. *P < .05, **P < .01, ***P < .001.

To determine ID hierarchy, we performed HAI against a panel of five mutant viruses where the classically defined H1 antigenic sites from A/Michigan/45/2015 had been substituted with heterologous antigenic sites from either H5 or H13 HAs: H1-ΔSa, H1-ΔSb, H1-ΔCb H1-ΔCa1 and H1-ΔCa2. Since these are both avian influenza viruses not endemic in humans, no specific antibody responses against these sites were expected in human sera. A significant reduction in HAI titres against a specific virus compared to Wt indicated loss of activity directed against the mutated antigenic site and therefore presence of antibodies in serum targeting such antigenic site. Main strategy of virus generation was described by Liu et al. [18] and is also illustrated in Supplementary Figure S1.

### Baseline immunodominance landscape

**Figure 1.**
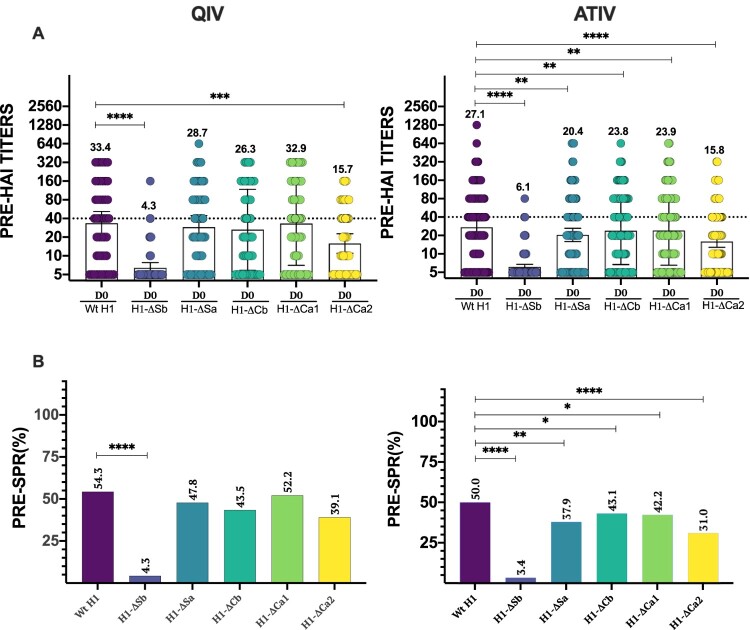
Baseline antibody levels. In (A) individual profiles of HAI antibody responses at day 0 against Wt H1 and modified viruses H1-ΔSb, H1-ΔSa, H1-ΔCb and H1-ΔCa1 and H1-ΔCa2 is represented. GMT value is marked in each column. *P*-values were determined with the repeated measures one-way Bonferroni’s ANOVA for multiple comparisons test with the Geisser–Greenhouse correction; *P < .05, **P < .01, ***P < .001, ****P < .0001. In (B) SPR before vaccination was calculated as the percentage of patients that achieved HAI titres ≥ 1/40 for each virus and compared to the respective Wt H1 for each cohort. The two-tailed *P*-value was calculated with the McNemar’s test with the continuity correction; *P < .05, **P < .01, ***P < .001, ****P < .0001.

To characterize the ID hierarchy at baseline before vaccination, we investigated the levels of anti-HA antibodies by HAI assays against the panel of mutant viruses and against the wild type (Wt) H1 as the reference strain considered homologous to the pandemic H1 strain in the vaccine. Since these are pre-existing antibodies before vaccination, the main difference between the QIV and the ATIV cohorts is age the QIV cohort including individuals younger than 65 years old, and the ATIV cohort having individuals older than 65 years old. Figure 1(A) shows HAI titres of each individual patient together with the calculated GMT and CI95% for each cohort at day 0. As expected, both cohorts had the highest GMT against the Wt H1 virus, with a value of 33.4 and 27.1 for the QIV and ATIV cohorts, respectively. When compared to Wt H1, the QIV cohort revealed significantly lower GMT at baseline against H1-ΔSb and H1-ΔCa2 indicating high levels of antibodies targeting these two antigenic sites. On the other hand, no significant reduction was observed when assessing the antibody response against the H1-ΔSa, H1-ΔCb and H1-ΔCa1. In addition, H1-ΔSb was significantly lower than H1-ΔCa2, revealing Sb as the most prevalent HA epitope in terms of amount of antibodies before vaccination. By contrast, the ATIV cohort showed a significantly lower basal GMT against not only H1-ΔSb and H1-ΔCa2, but also H1-ΔSa, H1-ΔCb and H1-ΔCa1 when compared to Wt H1 at baseline. Likewise, Sb remained the most immunodominant epitope, followed by Ca2. Those results confirmed that antibodies targeting both Sb and Ca2 are highly prevalent in the humoral antibody landscape against H1 IAV in adults. We next calculated the seroprotection rate (SPR, percentage with HAI titres ≥1/40) for each mutant virus and compared it to the H1-Wt SPR in each cohort (Figure 1(B)). Similar than GMT values, significant differences were found for H1-ΔSb compared to H1-Wt: 4.3% vs. 54.3% (*P* < .0001) in the QIV cohort while no significant differences were found when comparing the other viruses. On the other hand, SPR was significant different for all modified viruses when compared to Wt H1 in the ATIV cohort, particularly in the case of H1-ΔSb and H1-ΔCa2, with values of 3.4% and 31.0%, respectively, versus Wt H1 (50%)..

### Immunodominance after seasonal influenza vaccination

**Figure 2.**
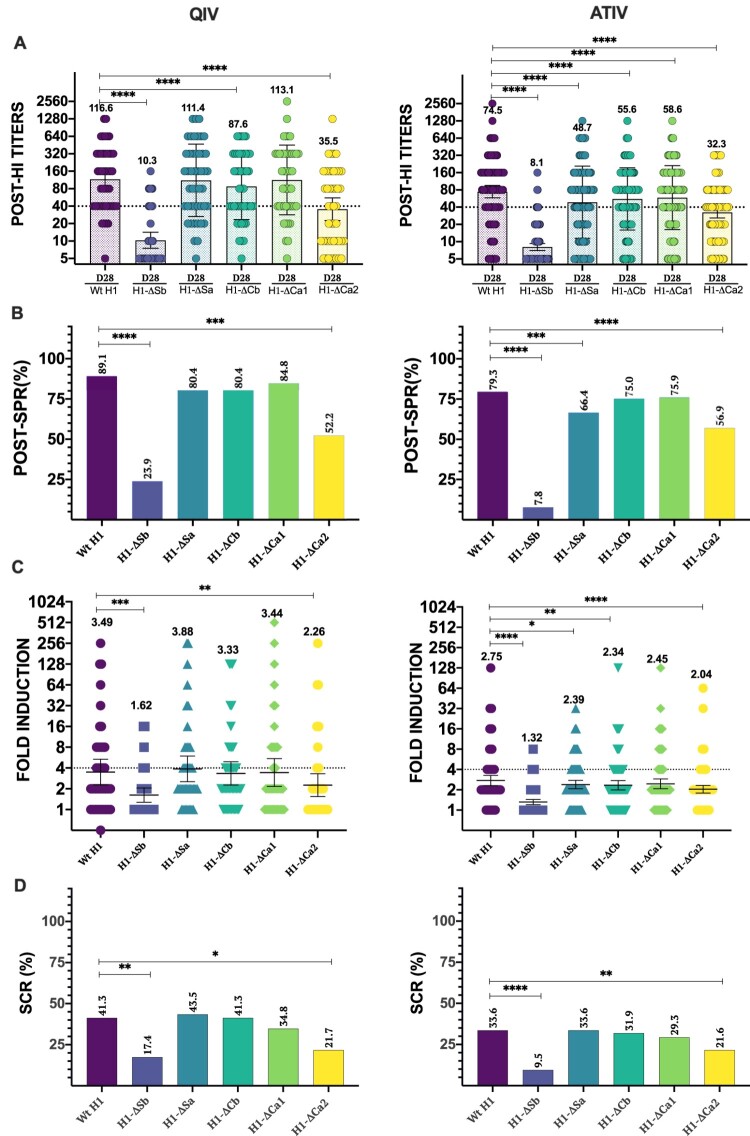
Response to vaccination. In (A) individual profiles of HAI antibody responses at day 28 against Wt H1 and modified viruses H1-ΔSb, H1-ΔSa, H1-ΔCb and H1-ΔCa1 and H1-ΔCa2 is represented. GMT value is marked in each column. *P*-values were determined with the repeated measures one-way Bonferroni’s ANOVA for multiple comparisons test with the Geisser–Greenhouse correction; *P < .05, **P < .01, ***P < .001, ****P < .0001. In (B) SPR after vaccination was calculated as the percentage of patients that achieved HAI titres ≥ 1/40 for each virus and compared to the respective Wt H1 for each cohort. The two-tailed *P*-value was calculated with the McNemar’s test with the continuity correction; *P < .05, **P < .01, ***P < .001, ****P < .0001. In (C) Fold induction of HAI titres or GMT increase (calculated as “GMTpost/GMTpre”) is represented of modified viruses H1-ΔSb, H1-ΔSa, H1-ΔCb and H1-ΔCa1 and H1-ΔCa2 compared to the Wt H1 in each cohort. *P*-values were determined with the repeated measures one-way Bonferroni’s ANOVA for multiple comparisons test with the Geisser–Greenhouse correction; *P < .05, **P < .01, ***P < .001, ****P < .0001. In (D) SCR was calculated as percentage of patients who reached a four-fold-induction for each virus and compared to its respective Wt H1 for each cohort. The two-tailed *P*-value was calculated with the McNemar’s test with the continuity correction; *P < .05, **P < .01, ***P < .001, ****P < .0001.

Overall, both vaccines produced a significant increase on HAI titres for all viruses except for H1-ΔSb. In QIV, a GMT of 116.6, 10.3, 111.4, 87.6, 113.1 and 35.5 was found for Wt, ΔSb, ΔSa, ΔCb, ΔCa1 and ΔCa2, respectively. Similar trend was found for ATIV, with GMT of 74.5, 8.1, 48.7, 55.6, 58.6 and 32.3 for Wt, ΔSb, ΔSa, ΔCb, ΔCa1 and ΔCa2, respectively (Supplementary Figure S2). Next, we calculated the SPR for each mutant virus. SPR was 89.1, 23.9, 80.4, 80.4, 84.8 and 52.2 for Wt, ΔSb, ΔSa, ΔCb, ΔCa1 and ΔCa2, respectively, in QIV. Meanwhile SPR was 79.3, 7.8, 66.4, 75.0, 75.9 and 56.9 for Wt, ΔSb, ΔSa, ΔCb, ΔCa1 and ΔCa2, respectively in ATIV (see Table 2). When compared to the Wt H1 in QIV differences were found. First, GMT values for H1-ΔSb, H1-ΔCa2 and exceptionally H1-ΔCb (Figure 2(A)) were significantly lower. However, only SPR against H1-ΔSb and H1-ΔCa2 were significantly lower compared to 89.1% against the Wt H1(Figure 2(B)). We next calculated fold induction (FI) from baseline levels and determine the seroconversion rates (SCR, percentage with four-fold induction) after vaccination in both cohorts. FI against the Wt H1 and the modified viruses are represented in Figure 2(C). Results showed significantly lower values against H1-ΔSb (1.62) and H1-ΔCa2 (2.26). Similarly, SCR represented in Figure 2(D) was 41.3% for Wt H1 virus, while significant lower values were found for H1-ΔSb and H1-ΔCa2, 17.4% and 21.7%, respectively. These results align with previous published data and highlight the immunodominant role of Sb and Ca2 in the influenza vaccine-induced antibody response. Similar trend was found for the ATIV cohort, although with some distinctions. While only differences in SPR were found for the H1-ΔSb and H1-ΔCa2 when compared to Wt H1 in the QIV, ATIV cohort also showed differences against the H1-ΔSa site. However, when comparing GMT values, a broader response was found and antibody titres against all antigenic sites showed a significant decrease compared to Wt H1 (Figure 2(A,B)). To understand if whether this broader response towards other classically less immunodominant antigenic sites such as Sa, Cb and Ca1, was due to pre-existing levels, we next calculated FI and SCR after ATIV vaccination. As shown in Figure 2(C,D) FI was significantly lower for all viruses except H1-ΔCa1. However, SCR was significantly lower for H1-ΔSb (9.5%) and H1-ΔCa2 (21.6%) compared to the Wt H1(33.6%). So far, these results showed the ID of Sb and Ca2 over other antigenic sites and suggest that the use of adjuvanted influenza vaccines could enhance the immunogenicity of less immunodominant antigenic sites. Next, we compared the response between different vaccine regimes (Table 2). Overall, no significant differences between vaccines were found in Wt H1. However, GMT values after vaccination were significantly reduced for H1-ΔSa, H1-ΔCb and H1-ΔCa1 for ATIV while GMT values for H1-ΔSb and H1-ΔCa2, remained similar in both cohorts. When comparing FI levels, a significant decrease was found for H1-ΔSa, H1-ΔCb and H1-ΔSb in the ATIV cohort, while SPR H1-ΔSb was also lower in the ATIV group. No differences between vaccines were found in SCR. The observed reduction of antibodies against additional antigenic sites in the ATIV cohort confirms the broader response induced by this vaccine against all antigenic sites compared to the QIV. To assess if differences in the magnitude of antibody responses according to type of vaccine could be biased by pre-existing immunity, we next investigated the SCR according to baseline seroprotection rates. As expected, previously non-seroprotected subjects presented higher SCR (Supplementary Figure S3). In the QIV group, the SCR was significantly higher in individuals non-seroprotected before receiving the vaccine for all viruses except for H1-ΔSb and H1-ΔCa2, where no significant differences depending on the previous protection status were found. In the case of the ATIV, SCR was significantly higher in previously non-seroprotected population for all viruses except for H1-ΔSb (*χ*^2^, *P* < .05).

**Table 2.** Vaccination responses in the QIV and ATIV cohorts.

VIRUS	POST-VACCINE	QIV	ATIV	*P*-value
Wt H1	GMTs (CI 95%)	116.6 (79.9–170.1)	74.5 (58.2–95.3)	.053
	SPR (%)	89.1	79.3	.142
	Fold induction	3.5	2.8	.218
	SCR (%)	41.3	33.6	.358
H1-ΔSb	GMTs (CI 95%)	10.3 (7.5–14.2)	8.1 (7.0–9.3)	.105
	SPR (%)	23.9	7.8	.005**
	Fold induction	1.6	1.3	.043*
	SCR (%)	17.4	9.5	.158
H1-ΔSa	GMTs (CI 95%)	111.4 (72.7–170.8)	48.7 (37.3–63.6)	<.001***
	SPR (%)	80.4	66.4	.077
	Fold induction	3.9	2.4	.007**
	SCR (%)	43.5	33.6	.240
H1-ΔCb	GMTs (CI 95%)	87.6 (59.2–129.6)	55.6 (44.1–69.9)	.042*
	SPR (%)	80.4	75.0	.462
	Fold induction	3.3	2.3	.043*
	SCR (%)	41.3	31.9	.256
H1-ΔCa1	GMTs (CI 95%)	113.1 (75.0–170.7)	58.6 (46.3–74.3)	.005**
	SPR (%)	84.8	75.9	.213
	Fold induction	3.4	2.5	.089
	SCR (%)	34.8	29.3	.497
H1-ΔCa2	GMTs (CI 95%)	35.5 (22.6–55.6)	32.3 (25.9–40.2)	.675
	SPR (%)	52.2	56.9	.585
	Fold induction	2.3	2.0	.515
	SCR (%)	21.7	21.6	.275

*P*-values were determined with the Student *T*-test for GMT and Fold-induction and chi-square test for SPR and SCR. *P < .05, **P < .01, ***P < .001.

### Immunodominance representation

**Figure 3.**
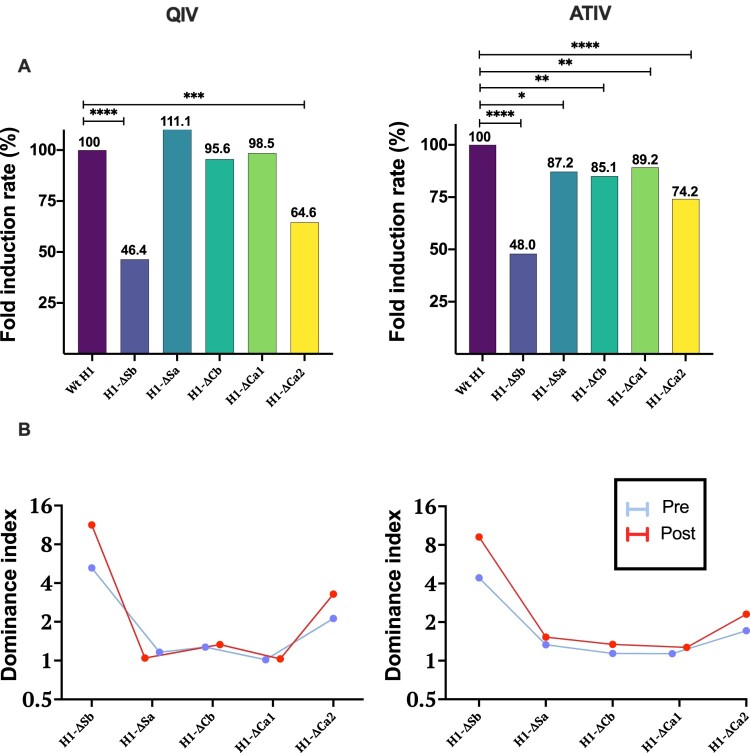
In (A) Fold Induction Rate ((FI mutant virus/FI Wt H1) × 100) of each virus is represented and compared to the Wt, which is 100%, in both cohorts. *P*-values were determined with the repeated measures one-way Bonferroni’s ANOVA for multiple comparisons test with the Geisser–Greenhouse correction; *P < .05, **P < .01, ***P < .001, ****P < .0001. In (B) Dominance index of HAI titres before and after vaccination with QIV and ATIV vaccines is represented.

Last, and to better illustrate the impact of the type of vaccine on the ID, we calculated two parameters: fold induction rate (FIR) and HAI dominance index (DI) (Figure 3). The FIR represents the percentage of FI achieved by each mutant virus, when considering Wt H1 as 100%. The second (DI), represents the reduction of HAI titres before and after vaccination in a mutant virus compared to its respective Wt H1(GMT Wt H1/GMT mutant virus) [18]. Therefore, higher values represent higher immunodominance. Significant differences were found in H1-ΔSb and H1-ΔCa2 with FIR of 46.4% and 64.6% after QIV vaccination. On the other hand, ATIV showed significant differences for all mutant viruses compared, with stronger reductions in the case of H1-ΔSb (48%) followed by H1-ΔCa2 (74.2%) (Figure 3(A)). When represented as HAI DI, responses following QIV vaccination were mainly generated against the dominant antigenic sites Sb and Ca2. Whereas, ATIV vaccination showed a broader immune response, despite a predominance of Sb (Figure 3(B)).

## Discussion

Our study demonstrates that ID can evolve under the influence of different factors. The term “Immunodominance” was first coined in 1966 [21]. ID describes the strong tendency of the immune response to respond to complex antigens in a hierarchical manner, with higher involvement of “immunodominant” antigens that potentially suppress responses to “subdominant” antigens [8]. Recently, the stem of the HA protein of influenza viruses has shown potential as a universal influenza vaccine candidate because it is very conserved among different subtypes of the same phylogenetic group. However, and despite being subjected to 2.2–2.4 times higher evolution rate than the stem domain, the head is the main target of neutralizing Abs, because of its ID over other more conserved epitopes [14,22,23]. Most vaccines elicit protective Abs and memory B cells that will respond quickly to a subsequently exposure. This can be related to serum HAI titres as a major correlate of protection against influenza-related illness [24,25]. Although other non-classical epitopes have been defined as HAI susceptible, in this work we have focused on characterization of the classically defined antigenic sites (Sa, Sb, Cb, Ca1 and Ca2) through HAI [15,16,26,27].

The influence of age in immune responses and vaccination has been known for many years [28–32]. In this study, two different cohorts are presented based on age as well as type of vaccine received. Our results show that there are already differences in the ID hierarchy before vaccination. The cohort receiving the QIV, which was significantly younger, showed a significant reduction of pre-vaccination HAI titres against H1-ΔSb and H1-ΔCa2 compared to the Wt H1, revealing an ID of Sb > Ca2 over the other antigenic sites. However, the cohort receiving the ATIV, formed by the elderly, showed a significant reduction in HAI titres against all mutant viruses, revealing an HAI ID of Sb > Ca2 > Sa > Cb > Ca1. Those findings suggest that age which can be considered a proxy of previous history of IAV exposures, is a factor that influences ID hierarchy of different antigenic sites. Young adults showed Sb and Ca2 as more immunodominant epitopes, whereas in the elderly responses also targeted other subdominant epitopes such as Sa, Cb and Ca1. The elderly have been, in general, more exposed to diverse and multiple influenza virus antigens, by both infection and vaccination. This could explain that subsequent exposures lead to a broader response against all epitopes. It is well known that Ab responses can be influenced by Ab competition for the same site and subsequent binding can be positively or negatively affected by allosteric effects [33]. We could therefore hypothesize that pre-existing Abs against more immunodominant epitopes saturate these sites, triggering responses directed to the subdominant epitopes in the elderly. In fact, there are previous studies in which the existence of high levels against dominant antigenic sites can influence Ab responses to those dominant sites by suppressing those responses in favour of subdominant sites after immunogen re-challenge [34]. Taking into consideration that both cohorts have comparable Ab levels, but responses differed, we suggest this could be the booster effect of adjuvants. On the other hand, younger adults have been less exposed by natural infection. Additionally, even though vaccination response tends to be inversely correlated with age, younger adults are not generally included in Spanish vaccination campaigns unless known risk factors for influenza disease severity are present. Thus, their response is more focused on more immunodominant epitopes which in the present study are Sb > Ca2. This is in line with previous antibody ID studies of the HA head epitopes indicating that humoral responses to infection differ from those induced by inactivated vaccines and major Ab responses upon vaccination is driven by prior exposures [8,11,35].

Responses to both vaccines were studied through FIR and HAI DI. Adjuvants have shown to improve vaccine performance, increasing humoral immune responses after seasonal influenza vaccination [36–41]. In fact, adjuvanted influenza vaccines have shown improved immunogenicity through higher HAI Abs titres and memory T and B cells against antigenically drifted influenza viruses [42]. In our study, the modification of the major antigenic sites results in a significant reduction of GMT values for H1-ΔSa, H1-ΔCb and H1-ΔCa1; and FIR for H1-ΔSb, H1-ΔSa and H1-ΔCb in the ATIV cohort. Those findings could suggest that ATIV increases the breath of protection by increasing the response towards all antigenic sites and not only the immunodominant ones. This aligns with the results found by Khurana et al. where the addition of MF59 adjuvant to standard vaccines expanded the Ab repertoire as well as increased avidity against HA head antigenic sites after vaccination [43]. Nonetheless, the influence of pre-existing immunity cannot be overruled in this group. On the other hand, with QIV showed a significant decrease of GMT only for H1-ΔSb and H1-ΔCa2, which might be explained by the lack of adjuvants in the vaccine or by individual differences. The above observations contrast with the responses found by Liu et al. where the HAI analysis against same epitopes in humans showed an ID profile of Sb, Sa > Ca1, Ca2, Cb. Liu et al. results align with recent studies in animals that appoint that the long-term response is dominated by antibodies against HA sites Sa and Sb [34]. However, experiments were performed only in 18 human donors [18], thus it is possible that ID hierarchy in humans differs from that of animals.

Our study has several limitations. First is the lack of data on cellular immune responses; however, this study was designed as a sero-epidemiological study on vaccine responses and only serum samples were available. Second, the cohorts analysed differ in age and therefore type of vaccine recommended by the Spanish Health agencies. That limited our conclusions on the impact of age on the immune response observed. However, the data on pre-existing immunodominance before vaccination helped to clarify this. Additionally, the cohort who received the ATIV is larger because influenza vaccination campaigns in Spain are mainly addressed to the elderly or population with pre-existing conditions, leaving a small number of samples from healthy young adults. In addition, as previous exposures to influenza virus influence responses after vaccination, different locations with different influenza circulation patterns could result in different ID hierarchy responses. Further studies will be needed to improve our understanding of the mechanisms involved in ID after influenza vaccination and its correlation with influenza disease protection.

## Supplementary Material

Supplemental Material

## Data Availability

The data that support the findings of this study are available from the corresponding authors, T.A. and A.G-S. upon reasonable request.

## References

[1] Krammer F, Smith GJD, Fouchier RAM, et al. Influenza. Nat Rev Dis Primers. 2018;4:1–21. 29955068 10.1038/s41572-018-0002-y PMC7097467

[2] WHO. Fact Sheets – Seasonal Influenza. [accessed 2020 Jul 5]. Available from: https://www.who.int/es/news-room/fact-sheets/detail/influenza-(seasonal).

[3] Paget J, Spreeuwenberg P, Charu V, et al. Global mortality associated with seasonal influenza epidemics: new burden estimates and predictors from the GLaMOR project. J Glob Health. 2019;9:1–12. 10.7189/jogh.09.020421 PMC6815659 31673337

[4] Preaud E, Durand L, Macabeo B, et al. Annual public health and economic benefits of seasonal influenza vaccination: a European estimate. BMC Public Health. 2014;14:1–12. 25103091 10.1186/1471-2458-14-813 PMC4141103

[5] Molinari NAM, Ortega-Sanchez IR, Messonnier ML, et al. The annual impact of seasonal influenza in the US: measuring disease burden and costs. Vaccine. 2007;25:5086–5096. 17544181 10.1016/j.vaccine.2007.03.046

[6] Belongia EA, Skowronski DM, McLean HQ, et al. Repeated annual influenza vaccination and vaccine effectiveness: review of evidence. Expert Rev Vaccines. 2017;16:723–736. 10.1080/14760584.2017.1334554 28562111

[7] Yu X, Tsibane T, McGraw PA, et al. Neutralizing antibodies derived from the B cells of 1918 influenza pandemic survivors. Nature. 2008;455:532–536. 18716625 10.1038/nature07231 PMC2848880

[8] Altman MO, Angeletti D, Yewdell JW. Antibody immunodominance: the key to understanding influenza virus antigenic drift. Viral Immunol. 2018;31:142–149. 29356618 10.1089/vim.2017.0129 PMC5863095

[9] Yu ED, Grifoni A, Sutherland A, et al. Balanced cellular and humoral immune responses targeting multiple antigens in adults receiving a quadrivalent inactivated influenza vaccine. Vaccines (Basel). 2021;9. doi:10.3390/vaccines9050426. PMC8146362 33922875

[10] Nelson MI, Holmes EC. The evolution of epidemic influenza. Nat Rev Genet. 2007;8:196–205. 17262054 10.1038/nrg2053

[11] Krammer F. The human antibody response to influenza A virus infection and vaccination. Nat Rev Immunol. 2019;19:383–397. 30837674 10.1038/s41577-019-0143-6

[12] Aydillo T, Escalera A, Strohmeier S, et al. Pre-existing hemagglutinin stalk antibodies correlate with protection of lower respiratory symptoms in Flu-infected transplant patients. Cell Rep Med. 2020;1. doi:10.1016/J.XCRM.2020.100130. PMC7691380 33294855

[13] Jegaskanda S, Andrews SF, Wheatley AK, et al. Hemagglutinin head-specific responses dominate over stem-specific responses following prime boost with mismatched vaccines. JCI Insight. 2019;4. doi:10.1172/jci.insight.129035. PMC6948858 31723058

[14] Angeletti D, Yewdell JW. Understanding and manipulating viral immunity: antibody immunodominance enters center stage. Trends Immunol. 2018;39:549–561. 29789196 10.1016/j.it.2018.04.008

[15] Caton AJ, Brownlee GG, Yewdell JW, et al. The antigenic structure of the influenza virus A/PR/8/34 hemagglutinin (H1 subtype). Cell. 1982;31:417–427. 6186384 10.1016/0092-8674(82)90135-0

[16] Gerhard W, Yewdell J, Frankel ME, et al. Antigenic structure of influenza virus haemagglutinin defined by hybridoma antibodies. Nature. 1981;290:713–717. 6163993 10.1038/290713a0

[17] Sriwilaijaroen N, Suzuki Y. Molecular basis of the structure and function of H1 hemagglutinin of influenza virus. Proc Jpn Acad Ser B Phys Biol Sci. 2012;88:226–249. 10.2183/pjab.88.226 PMC3410141 22728439

[18] Liu STH, Behzadi MA, Sun W, et al. Antigenic sites in influenza H1 hemagglutinin display species-specific immunodominance. J Clin Invest. 2018;128:4992–4996. 30188868 10.1172/JCI122895 PMC6205383

[19] Erbelding EJ, Post DJ, Stemmy EJ, et al. A universal influenza vaccine: the strategic plan for the national institute of allergy and infectious diseases. J Infect Dis. 2018;218:347–354. 29506129 10.1093/infdis/jiy103 PMC6279170

[20] WHO Global Influenza Surveillance Network. Manual for the laboratory diagnosis and virological surveillance of influenza. Geneva: WHO Press; 2011.

[21] Lüderitz O, Staub AM, Westphal O. Immunochemistry of O and R antigens of Salmonella and related Enterobacteriaceae. Bacteriol Rev. 1966;30:192–255. 5324647 10.1128/br.30.1.192-255.1966 PMC378223

[22] Kirkpatrick E, Qiu X, Wilson PC, et al. The influenza virus hemagglutinin head evolves faster than the stalk domain. Sci Rep. 2018;8:10432. 29992986 10.1038/s41598-018-28706-1 PMC6041311

[23] Mathew NR, Angeletti D. Recombinant influenza vaccines: saviors to overcome immunodominance. Front Immunol. 2020;10:1–10. 10.3389/fimmu.2019.02997 PMC6966699 31998299

[24] Plotkin SA. Correlates of protection induced by vaccination. Clin Vaccine Immunol. 2010;17:1055–1065. 20463105 10.1128/CVI.00131-10 PMC2897268

[25] Ohmit SE, Petrie JG, Cross RT, et al. Influenza hemagglutination-inhibition antibody titer as a correlate of vaccine-induced protection. J Infect Dis. 2011;204:1879–1885. 21998477 10.1093/infdis/jir661

[26] Lubeck MD, Gerhard W. Topological mapping of antigenic sites on the influenza A/PR/8/34 virus hemagglutinin using monoclonal antibodies. Virology. 1981;113:64–72. 6791373 10.1016/0042-6822(81)90136-7

[27] Matsuzaki Y, Sugawara K, Nakauchi M, et al. Epitope mapping of the hemagglutinin molecule of A/(H1N1)pdm09 influenza virus by using monoclonal antibody escape mutants. J Virol. 2014;88:12364–12373. 25122788 10.1128/JVI.01381-14 PMC4248900

[28] Fulop T, Larbi A, Dupuis G, et al. Immunosenescence and inflamm-aging as two sides of the same coin: friends or foes? Front Immunol. 2018;8; doi:10.3389/fimmu.2017.01960. PMC5767595 29375577

[29] Pera A, Campos C, López N, et al. Immunosenescence: implications for response to infection and vaccination in older people. Maturitas. 2015;82:50–55. 26044074 10.1016/j.maturitas.2015.05.004

[30] Sambhara S, McElhaney JE. Immunosenescence and influenza vaccine efficacy. Curr Top Microbiol Immunol. 2009;333:413–429. 19768417 10.1007/978-3-540-92165-3_20 PMC7121450

[31] Pawelec G. Age and immunity: what is “immunosenescence”? Exp Gerontol. 2018;105:4–9. 29111233 10.1016/j.exger.2017.10.024

[32] Makinodan T. Nature of the decline in antigen-induced humoral immunity with age. Mech Ageing Dev. 1980;14:165–172. 7010008 10.1016/0047-6374(80)90115-3

[33] Angeletti D, Yewdell JW. Is it possible to develop a “universal” influenza virus vaccine? outflanking antibody immunodominance on the road to universal influenza vaccination. Cold Spring Harb Perspect Biol. 2018;10:1–9. 10.1101/cshperspect.a028852 PMC6028072 28663210

[34] Angeletti D, Gibbs JS, Angel M, et al. Defining B cell immunodominance to viruses. Nat Immunol. 2017;18:456–463. 28192417 10.1038/ni.3680 PMC5360521

[35] Kim JH, Liepkalns J, Reber AJ, et al. Prior infection with influenza virus but not vaccination leaves a long-term immunological imprint that intensifies the protective efficacy of antigenically drifted vaccine strains. Vaccine. 2016;34:495–502. 26706277 10.1016/j.vaccine.2015.11.077 PMC4713344

[36] O’Hagan DT, Ott GS, Van NG, et al. The history of MF59® adjuvant: a phoenix that arose from the ashes. Expert Rev Vaccines. 2013;12:13–30. 23256736 10.1586/erv.12.140

[37] O’Hagan DT, Rappuoli R, De Gregorio E, et al. MF59 adjuvant: the best insurance against influenza strain diversity. Expert Rev Vaccines. 2011;10:447–462. 21506643 10.1586/erv.11.23

[38] Ko E-J, Lee Y-T, Kim K-H, et al. Effects of MF59 adjuvant on induction of isotype-switched IgG antibodies and protection after immunization with T-dependent influenza virus vaccine in the absence of CD4 + T cells. J Virol. 2016;90:6976–6988. 27226368 10.1128/JVI.00339-16 PMC4944285

[39] Della Cioppa G, Nicolay U, Lindert K, et al. Superior immunogenicity of seasonal influenza vaccines containing full dose of MF59® adjuvant. Hum Vaccin Immunother. 2012;8:216–227. 22426371 10.4161/hv.18445

[40] Even-Or O, Samira S, Ellis R, et al. Adjuvanted influenza vaccines. Expert Rev Vaccines. 2013;12:1095–1108. 24053401 10.1586/14760584.2013.825445

[41] Galli G, Hancock K, Hoschler K, et al. Fast rise of broadly cross-reactive antibodies after boosting long-lived human memory B cells primed by an MF59 adjuvanted prepandemic vaccine. Proc Natl Acad Sci U S A. 2009;106:7962–7967. 19416838 10.1073/pnas.0903181106 PMC2674105

[42] Ko EJ, Kang SM. Immunology and efficacy of MF59-adjuvanted vaccines. Hum Vaccin Immunother. 2018;14:3041–3045. 30015572 10.1080/21645515.2018.1495301 PMC6343625

[43] Khurana S, Verma N, Yewdell JW, et al. MF59 adjuvant enhances diversity and affinity of antibody-mediated immune response to pandemic influenza vaccines. Sci Transl Med. 2011;3:85ra48. 10.1126/scitranslmed.3002336 PMC3501657 21632986

