# Supplementary material for: Immunodominance hierarchy after seasonal influenza vaccination
Source: Emerg Microbes Infect. 2022 Nov 4;11(1):2670–9. doi: 10.1080/22221751.2022.2135460 (PMC9639523; doi:10.1080/22221751.2022.2135460)
Supplement: Supplemental Material [file TEMI_A_2135460_SM3049.docx]

**Supplementary Figure 1**. In **A** Crystal structure of the HA trimer of the pandemic H1 A/ Michigan/45/2015H1 strain (PDB:3UBE) (top view and side view) The receptor binding site (RBS) is colored in pink. Classically defined antigenic sites are in colors. Modeling was performed with PyMOL (The PyMOL Molecular Graphics System, Version 2.5.1, Schrödinger, LLC). In **B** Amino acid sequence of A/Michigan/45/2015. Original sequences of head domain antigenic sites are in white squares ﻿and described below on the left. Amino acid sequences of epitope substitutions of mutant viruses are in colored squares on the right corresponding to the colors in the crystal structure in 1A.

**Supplementary Figure 2**. **Increase of HAI titres.** Individual values of HAI titers and the GMT(CI95%) for each virus before and after vaccination is represented. P values were calculated with Friedman´s test applying Dunn´s correction; *P<0.05, **P<0.01, ***P<0.001, ****P<0.0001.

**Supplementary figure 3**. Comparison of the response to vaccination of non-seroprotected vs. seroprotected population for each virus is represented for QIV and for ATIV. Seroconversion is described in each column and represents the percentage of population that has reached a four-fold-induction of HI titres. The two-tailed P values were calculated with using chi-square test; *P<0.05, **P<0.01, ***P<0.001, ****P<0.0001
